# Phyto‐fabricated silver nanoparticles inducing microbial cell death via reactive oxygen species‐mediated membrane damage

**DOI:** 10.1049/nbt2.12036

**Published:** 2021-04-21

**Authors:** L. Srinivas Naik, Ch. Venkata Ramana Devi

**Affiliations:** ^1^ Department of Biochemistry University College of Science Osmania University Hyderabad India

## Abstract

Eco‐friendly synthesis of the silver nanoparticles (AgNPs) has a number of advantages like simplicity, biocompatibility, low toxicity in nature over their physical and chemical methods. In the present study, the authors report biosynthesized AgNPs using the root extract of the perennial plant ‘Spiny gourd’ (*Momordica dioica)* and investigated their anti‐bacterial application with mechanistic approaches. Different biophysical techniques such as UV‐Vis spectroscopy, FTIR, XRD, TEM, SAED, and DLS were employed for AgNPs characterization. The synthesized AgNPs were polydispersed, crystalline in nature, with anionic surface (−22.3 mV), spherical in shape with an average size of 13.2 nm. In addition, the AgNPs were stable in room temperature and in different biological buffers. The anti‐bacterial activities of AgNPs were studied with respect to the pathogens such as *Bacillus subtilis*, *Staphylococcus aureus* (Gram‐positive), *Pseudomonas aeruginosa*, *Escherichia coli, Klebsiella planticola* (Gram‐negative), and *Candida albicans*. Also, mechanistic studies of AgNPs such as protein leakage assay, nucleic acid leakage assay, ATP leakage assay, ROS accumulation, determination of biofilm degrading activity, measurement of potassium, showing that the synthesized AgNPs are capable of containing a potential application in the antimicrobial therapeutic agents and the pharmaceutical industry.

## 1 INTRODUCTION

Nanotechnology is one of the major promising and latest research areas in the current science and technology field. Nanoparticles with new and better properties were synthesized based on shapes and sizes having potential applications in health care, catalysis, cosmetics, biosensing [1], drug delivery [2], environmental [3], anti‐bacterial [4], anti‐inflammatory [5], anti‐cancer [6], and biomedical applications, and so forth [7]. The green synthesis of AgNPs is essential as the other therapeutic uses [8]. Frequently a number of methods have been used in the synthesis of AgNPs which includes physical and chemical methods [9], thermolysis [10], microwave assisted [11], photochemical reduction [12], and phytosynthesis [13], and chemical reduction [14]. Chemically synthesized nanoparticles are known to have high toxicity due to various chemicals used and are thus environmentally unfriendly [15]. Bio‐fabrication methods for synthesis of nanoparticle using plant extract and microorganisms [16] have been extensively developed for nanoparticle synthesis which is advantageous over physico‐chemical methods. These eco‐friendly methods are cost effective, non‐toxic, reliable and can be simply scaled up for huge scale synthesis of stable nanoparticles [17].

Frequent infections by microbes such as bacteria, yeasts, viruses, and moulds affect the human health regularly due to environmental exposure. Emergence of number of multi drug‐resistant microbes stresses the importance of developing new antimicrobial agents that can overcome drug resistance and are cost effective simultaneously. This led to the use of silver‐based antiseptics with broad‐spectrum antimicrobial activity which also have a significantly lower tendency to induce resistance compared to conventional antibiotics [18]. It was reported earlier that silver ions exert strong anti‐bactericidal or inhibitory effects in addition to possessing a broad spectrum of antimicrobial activities [19]. Silver ions affect cell membranes of bacteria which are associated with many enzymes and proteins by releasing K^+^ ions thus, showing antibacterial properties [20]. Thus, cell membrane is a potential target site for silver ions [21]. Silver ions get accumulated in the vacuole and cell walls and other cellular contents in the form of granules leading to structural abnormalities and thus causing inhibition of bacterial growth [22]. Silver ions also interact with the nucleic acids where they interact specifically with bases in the DNA helix rather than with phosphate group, although the role of this mechanism in anti‐bacterial effect is not clearly understood [23].

Previous studies reported that the antibacterial activity of AgNPs may be due to the involvement of free radicals such as reactive oxygen species (ROS) which can affect the bacterial DNA or mitochondria, even though the mechanism of action is inadequately understood [24]. Some AgNPs produce ROS such as superoxide anion (O^2*−*^), singlet oxygen (_1_O^2^) and hydroxyl radical (OH^*·*^) which in turn cause oxidative damage resulting in good antibacterial effects [25]. Traditionally*, Escherichia coli* and *Staphylococcus aureus* are widely used to test the anti‐bacterial mechanism studies. *E. coli* and *S. aureus* are mostly found on the skin and digestive tract of mammals and often cause diseases. These strains are chosen due to their unique cell membrane characteristics because one is Gram negative and positive [26].

Hence the aim of the present study is to develop a green approach for the synthesis of AgNPs using root extract of the creeper plant *Momordica dioica* [27]. *M. dioica* is a well‐recognized therapeutic plant belonging to the cucurbitaceae family which originate in tropical and subtropical regions of the Earth. *M. dioica* has several applications in the treatment of stomach pain, diabetes, fevers, leprosy, snake bite, cancer, menstrual disorders, infections, and hypertension and shows anti‐allergic, anti‐bacterial, anti‐cancer, anti‐helminthic, and anti‐scavenging properties [28, 29]. Not much literature is available about the pharmacological and other activities on this plant. In the present work, we synthesized silver nanoparticles (AgNPs) using an aqueous extract of the root of *M. dioica* and silver nitrate under sunlight [30]. Further, these nanoparticles are characterized by different techniques such as UV‐Visible spectroscopy, FTIR, XRD, TEM, SAED, and DLS. The optimization for biosynthesis of AgNPs was done by varying concentrations of root extract, metal ions, and time of sunlight exposure. The synthesized AgNPs displayed good anti‐bacterial and anti‐fungal activities against the selected pathogens such as *Bacillus subtilis* ATCC 6051, *S. aureus* ATCC 29,736 (Gram‐positive), *Pseudomonas aeruginosa* ATCC 27,858, *E. coli* ATCC 8739*, Klebsiella planticola* ATCC 2719 (Gram‐negative), and *Candida albicans* ATCC 2091. Mechanistic studies such as protein leakage assay, nucleic acid leakage assay, ATP leakage assay, ROS accumulation, determination of biofilm degrading activity and measurement of potassium, show that the synthesized AgNPs are capable of containing the potential application in antimicrobial therapeutic agents and pharmaceutical industry.

## 2 MATERIAL AND METHODS

Silver nitrate (AgNO_3_), Mueller Hinton Broth (MHB), Tryptic soy broth (TSB), Potato dextrose broth (PDB), and other culture media used for microorganisms were purchased from HiMedia Laboratories Pvt Ltd, Mumbai, India.

### 2.1 Procurement and identification of plant source

Healthy roots of the perennial plant, ‘Spiny gourd’ (*M. dioica)*, were collected from the forest region near Lalapuram Village, Konijerala Mandal, Khammam District, Telangana (State), India. The collected roots were taxonomically identified and authenticated (Voucher No – 72) by the Assistant Professor, Mr. A. Vijaya Bhasker Reddy, Department of Botany, Nizam College, Osmania University, Hyderabad, Telangana, India.

### 2.2 Preparation of water extract of *M. dioica* root

The roots of *M. dioica* were cleaned thoroughly with Milli‐Q water to remove mud and air dried at room temperature (25 ± 3°C). Thus, dried roots were made into fine powder by crushing. Two grams of the root powder was suspended in 100 ml of Milli‐Q water and heated at 50°C for 15 min to obtain the watery extract of the root. Furthermore, unwanted solids in the extract were removed by filtering through Whatman filter paper (11 μm medium flow filter paper). The filtrate was centrifuged at 5000 rpm for 10 min and the supernatant was collected and stored at 4°C.

### 2.3 Biosynthesis of silver nanoparticles using *M. dioica* root extract

Biosynthesis of AgNPs was carried out by adding 900 ml of *M. dioica* root extract and 100 ml of 1 mM AgNO_3_ (aqueous). The reaction mixture was exposed to sunlight, and nanoparticle formation was identified by observing the colour change and characterized by UV‐Vis spectroscopy. The experiment was repeated in dark condition by carrying out the reaction in a brown coloured closed container at room temperature (25 ± 3°C). The reaction parameters such as time of sunlight exposure, concentration of root extract and the AgNO_3_ concentration were optimized. The root extract concentrations varied from 1.0% to 9.0% (v/v) and the AgNO_3_ concentration varied from 0.5 to 10 mM. The biosynthesized AgNPs were pelleted by centrifuging reaction mixture at 15,000 rpm for 20 min and washed with Milli‐Q water to remove debris. Thus, obtained pellet was washed thrice with Milli‐Q water and finally dried using vacuum drying and the AgNPs powder was stored at 4°C for further studies.

### 2.4 UV–Visible spectrophotometric analysis

The AgNPs biosynthesized using the aqueous root extract of *M. dioica* were analysed by UV–Visible spectrophotometer. Based on the particle shape, size, and size distribution, and surface plasmon resonance (SPR), the AgNPs exhibit the characteristic optical properties [31]. The excitation energy of the surface plasmonic vibrations due to reduction of Ag + ions was observed every 10 min using the UV‐Visible spectrophotometer. Absorption maxima of test sample were measured from 200 to 700 nm by using UV‐Visible spectrophotometer (ELICO SL‐159) after appropriate dilution.

### 2.5 Fourier‐transform infrared spectroscopy (FT‐IR)

The functional groups of the biosynthesized AgNPs were analysed using the FT‐IR spectroscopy. The dried nanoparticle powder is mixed with KBr to form a pellet and was used for FT‐IR analysis using Shimadzu IR‐IR affinity model. The FT‐IR spectroscope was operated at a resolution of 0.4 cm^−1^ within wave number region of 400–4000 cm ^−1^ [32].

### 2.6 X‐ray diffraction (XRD)

The crystallization of the biosynthesized AgNPs was studied by X‐ray diffraction data. The sample was held in a cavity within a glass slide. After fixing the AgNPs powder sample in the cavity, the glass slide was placed in the diffractometer. The data scan range of 2*θ* = 0–80° was used in the diffractometer which was controlled using the data scan software [33].

### 2.7 Transmission electron microscopy (TEM)

The morphological characterization of biosynthesized AgNPs was performed using the transmission electron microscopy (TEM). The AgNPs were subjected to ultrasonication, and a drop of the test sample was placed on top of carbon‐coated copper grids [34]. Technai‐FE 12 (Philips, Holland) is used to acquire TEM micrographs. The instrument was maintained at an accelerating voltage of 120 kV, and a vacuum of 10^−7^ Torr is applied. Diffraction pattern of sample was studied by observing central region of the specimen.

### 2.8 Dynamic light scattering (DLS)

The biosynthesized pulverized nanoparticle was processed for dynamic light scattering (DLS) measurements after ultrasonication and suspending in Milli‐Q water at 25°C to measure the particle size and surface charge using Zetasizer Nano (Malvern Instruments Ltd, UK) [35]. The size distribution range of the nanoparticles was determined by the polydispersity index (PDI)

### 2.9 Inductively coupled plasma atomic emission spectrometry (ICP‐AES)

The metal ion concentration (ppm) of AgNPs in the solution was determined using the ICP‐AES (ICP‐AES, IRIS Intrepid II XDL, Thermo Jarrel Ash, USA) and was calculated using AgNO_3_ standard graphs (1–50 ppm) plotted from ICP‐AES analysis [36].

### 2.10 In vitro stability and catalytic studies of silver nanoparticles

The stability of nanoparticles is an important parameter prior to use their biomedical application. The in vitro stability of green synthesized AgNPs was studied in different solutions such as water, foetal bovine serum (FBS), phosphate buffer (PBS), and 5% NaCl, 10% NaCl, buffered saline with pH 5.0, 7.0, and 9.0. The nanoparticle solution (800 µl) was added to each solution (200 µl) and incubated at room temperature for 0 h to 4 weeks. The stability of nanoparticles was evaluated by recording the optical density values at 445 nm for AgNPs [37]. The green synthesized AgNPs were used as catalytic activity by the reduction of 4‐nitrophenol (4‐NP) to 4‐aminophenol through sodium borohydride (NaBH_4_).

### 2.11 Antimicrobial activity of AgNPs

Different microbial strains *B. subtilis* ATCC 6051, *S. aureus* ATCC 29736 (Gram‐positive), *P. aeruginosa* ATCC 27858, *E. coli* ATCC 8739*, K. planticola* ATCC 2719 (Gram‐negative), and *C. albicans* ATCC 2091 were used in this study. All strains in the present study were procured from the American Type Culture Collection (ATCC) USA, stored at −80^o^C in glycerol (25% v/v), and were sub‐cultured whenever required for further studies.

### 2.12 The minimum inhibitory concentration (MIC) assay

The MIC assay of biosynthesized AgNPs was performed by broth microdilution method as per Clinical Laboratory Standards Institute (CLSI) using 96‐well microtitre plates [38]. The pathogenic microbial cultures were grown overnight in the MHB and PDB broth and diluted approximately to (10^6^ cfu ml^−1^) with a sterile medium. To these cultures, 100 µl of MHB broth with different concentrations (100, 50, 25, 12.5, 6.25, 3.12, 1.5, 0.78, 0.39, and 0.19 µg/ml) of root extract, and AgNPs were added. Miconazole was added as a positive control for *Candida* and ampicillin for bacteria, and incubated at 37^o^C. After 24 h incubation, 40 µL of the *p*‐iodonitrotetrazolium (INT) dye (0.02%, 20 mg INT dissolved in 100 ml of 40% dimethylformamide) was added to each well and incubated for 3 h. The concentration of *p*‐iodonitrotetrazolium which is reduced over time can be measured at 450 nm using ELISA reader to find the minimum inhibitory concentration (MIC). The minimum concentration or the lowest concentration required to completely inhibit the microbial growth was noted as MIC. These suggest that higher concentration of nanoparticle is required to kill microorganism. All the experiments were performed in triplicates.

### 2.13 Minimum bactericidal concentration (MBC) assay

The bactericidal assays were performed in 2.0 ml sterile micro centrifuge tubes. Pathogenic microbial strains were grown overnight in the MHB broth and adjusted to a concentration of (1.5 × 10^8^ cfu ml^−1^) sterile medium. AgNPs of different concentrations were added to 100 µl of above cultures in test tubes and were incubated at 37°C overnight. An aliquot (10 µl) of the bacterial suspensions from each tube was spread on MHA agar plates and incubated further at 37°C for 24 h and observed later for the growth. The MBC was determined as the lowest concentration of test compound essential to accomplish a reduction in bacterial cell number (killing). The MBC values are the mean of the values from triplicates [39].

### 2.14 Minimum fungicidal concentration (MFC) assay

The anti‐fungal activity was performed in sterile 2 ml micro centrifuge tubes. Pathogenic strains were cultured overnight in PDB broth and adjusted to inoculum concentration of (1.5 × 10^8^ cfu ml^−1^). Then AgNPs of different concentrations were added to 100 µl of the above cultures in test tubes and were incubated at 30°C for 24 h. An aliquot (10 µl) of the *Candida* suspensions from the tubes were spread on potato dextrose agar plates and further incubated at 30°C. After 24 h of incubation, the plates were observed for growth. The MFC was showed as the lowest concentration of test compound vital to kill a particular fungal strain. The MFC values are the mean of values from triplicates [40].

### 2.15 Determination of biofilm degrading activity

The biofilm degrading potential of biosynthesized AgNPs against *S. aureus*, *B. subtilis*, *K. planticola*, and *E. coli* was determined using the colourimetric method. Initially, the 100 μl of bacterial cultures in the log phase were added to the 96‐well microtitre plates. After 24 h of incubation, AgNPs of different concentrations (2 to 10 μg/ml) were added. Then plates were incubated for 4 h at 37°C. Later, each were harvested by low‐speed centrifugation at 4°C and washed thrice with 200 μl of sterile water. The microtitre plate was air dried in aseptic conditions for 45 min. Then, 100 μl of crystal violet solution (0.1% v/v*)* was added to every well and incubated for 30 min at room temperature. The excess dye was washed thrice with 200 μl sterile water. The dye incorporated into the adhered cells was dissolved using 200 μl of 95% (v/v*)* ethanol. The concentration of the solubilized dye was determined using the absorbance at 595 nm, by the microplate ELISA reader. The percentage (%) of biofilm inhibition was calculated using the following formula: [1 − (test OD 595/control OD 595)] × 100 [41]. The experiments were performed in thrice and data was interpreted in terms of mean ± SD.

### 2.16 Mechanism of action of silver nanoparticles on microbial cells―killing kinetics of microbes

The bacterial in vitro time‐kill assay was determined for AgNPs against the pathogenic bacteria, *S. aureus* and *E. coli*. At first, the bacterial cells were grown in the MHB broth up to mid‐logarithmic phase. Different concentrations of green synthesized AgNPs in PBS were added to the above cultures with final concentration of (10^7^ cfu/ml) and were incubated 24 h in shaking incubator (300 rpm) at 37°C. Samples were collected at 0, 1, 2, 4, 6, and 8 h after addition of AgNPs and spread on MH agar plates for observation of growth. After incubation the microbial colonies were counted by colony counter and recorded. The cultures which are not exposed to AgNPs were taken as control, and the experiments were carried out in three independent experiments [39].

### 2.17 Measurement of protein leakage assay

The intracellular protein leakage assay was carried out as per the method explained by [42]. Bacterial cultures were treated with suitable MIC concentrations of AgNPs solution for up to 4 and 8 h. After incubation, the whole cultures broth was centrifuged at 6000 rpm for 20 min to remove debris and the supernatants were collected. For each test sample, 200 μl of the supernatant was mixed with 800 μl of Bradford reagent and incubated for 10 min at room temperature. The OD was noted at 595 nm using UV‐Visible spectrophotometer to determine the concentration of leaked protein in the supernatant. Bovine serum albumin (BSA) was used as a standard and each experiment was repeated three times.

### 2.18 Nucleic acid leakage assay

The nucleic acid leakage assay was performed according to the procedure given by [43] with slight changes. Aliquots of 3 ml of overnight grown bacterial cultures were treated with AgNPs up to different time intervals that is, 4 and 8 h. Then, the suspensions were filtered using Millex‐GS syringe filter having 25 mm diameter and 0.2 μm pore size (Millex‐GS, Millipore, and Madrid, Spain). Quantification of nucleic acids in the filtrate was done by determining the absorbance at 260 nm using the UV‐Vis spectrophotometer. The results were validated by repeating the experiment for three times.

### 2.19 ATP leakage assay

The ATP leakage from the bacterial cells (*S. aureus* and *E. coli*) was determined by using bioluminescence assay [44]. Initially, the pathogenic microorganisms were grown in MHB broth at 37°C for 24 h and subsequently incubated with an appropriate MIC of AgNPs. Samples at different time intervals of 0, 5, 10, 20, 30, and 60 min were collected and bacterial cells were collected by centrifugation at 5000 rpm for 10 min. Later, the cells were washed twice with buffers 50 mM potassium phosphate (pH 7.0) and 50 mM HEPES (pH 7.0). An aliquot (150 µL) of the supernatant was mixed with 75 µl of buffer (1.02% KH_2_PO_4_ + 0.143% MgCl_2_ + 0.0095% phosphoenol pyruvate, pH 7.3). Extracellular ATP and total ATP concentrations were determined using the luminometer (Novaspec II visible spectrophotometer, Pharmacia Biotech). The ATP leakage kinetics was measured for each bacterial suspension as explained above. ATP leakage assay and killing kinetics study was conducted under similar conditions. All the solutions used in the experiments were prepared using the ATP‐free water. Each experiment was performed triplicate.

### 2.20 Measurement of potassium

The measurement of intracellular K^+^ leakage from bacterial cells (*S. aureus* and *E. coli*) was measured by the inductively coupled plasma spectrometry (ICP) method using the Optima 3000 DV spectrometer (Perkin‐Elmer Corporation, Norwalk, USA). Approximately (1 × 10^5^) bacterial cells were washed twice with cold choline buffer and then suspended in same buffer and cell density was measured at 600 nm. Then, the bacterial cells were exposed to AgNPs. The nanoparticle‐induced K^+^ leakage was quantitatively measured by using ICP‐MS [45].

### 2.21 Detection of reactive oxygen species (ROS)

The influence of AgNPs on intracellular ROS accumulation was studied using 2′, 7′‐ dichlorofluorescein diacetate (DCFDA). The pathogenic bacteria cells were initially treated with suitable MIC of AgNPs and cell density of *E. coli* and *S. aureus* was adjusted to (1 × 10^5^ cfu ml). Similarly, negative control and positive control were assessed using untreated cells and hydrogen peroxide (H_2_O_2_ – 1 mM), respectively. These cells were incubated at 37^ο^C for 4 and 8 h. Later, the cultures were centrifuged at 4^o^C for 30 min at 3000 rpm to collect supernatant. The 100‐μM DCFDA was added to each supernatant and incubated for 1 h. The concentration of ROS accumulated in the supernatant was measured using an Infinite M200Pro (Tecan) microtitre plate reader where, 485 nm as fluorescence excitation wavelength and 528 nm as emission wavelength were used [46]. The experiments were repeated thrice, and the data is represented as mean ± SD.

## 3 RESULTS AND DISCUSSION

### 3.1 Biosynthesis of AgNPs using *M. dioica* root extract

The biological synthesis of AgNPs was confirmed by the colour change in the reaction mixture from pale yellow to brown which is due to the reduction of Ag^+^ ions. The reaction mixture which was exposed to sunlight exhibited a change in colour within 10 min of incubation, whereas the reaction mixture kept in the dark did not show any colour change even after 24 h of incubation. The formation of AgNPs was further confirmed by the sharp surface plasmon resonance (SPR) band at a wavelength of 445 nm. The faster biosynthesis of nanoparticles may be due to the exposure of sunlight. Jyoti et al. reported similar SPR band results for AgNPs synthesized using *Urtica dioica* leaves at 450 nm [47].

### 3.2 Optimized production of AgNP synthesis by aqueous root extract of *M. dioica*

**FIGURE 1:**
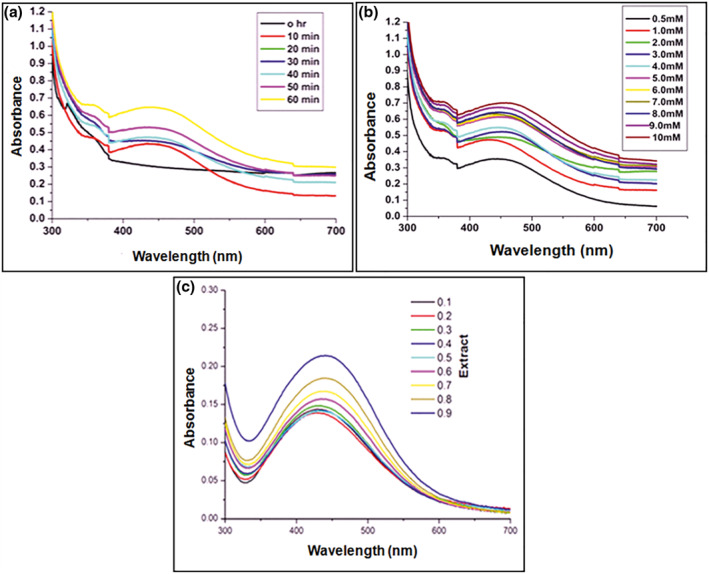
UV‐Visible spectra of green synthesized AgNPs at different (a) sunlight exposure time interval (b) concentrations of aq. AgNO_3_ and (c) concentrations of root extract

The effective therapeutic applications of nanoparticles depend on factors such as stability, shape, and size of the nanoparticles. Hence we employed varied conditions for the optimal synthesis of AgNPs by the root extract of *M. dioica* such as duration of sunlight exposure, concentrations of metal ions, and root extract concentrations. The different time intervals of sunlight exposure used in the present study were 10 to 60 min (Figure 1a). With increase in exposure time, there was an increase in the intensity of SPR band width along with a change in colour pattern which indicates reduction of AgNPs from Ag^+^ to Ag^0^. A sharp peak was observed at 445 nm in the sample exposed to 10 min of sunlight, which is the optimal time of incubation [48]. The samples exposed up to 60 min showed high peak intensities when compared to those exposed to lower time intervals.

Further optimization for nanoparticle synthesis was carried out by varying the metal ion concentrations (0.5 to 10 mM), and it was found that the optimum concentration for nanoparticle synthesis was 1 mM (Figure 1b). The sun light exposure time and root extract concentration were unchanged. With increase in the metal ion concentration, the availability of biological factors like polyphenols, flavonoids, terpenoids, and enzymes decreases [49]. Among the studied root extract concentrations (100 to 900 µl) for the synthesis of AgNPs, 100 µl/ml was found as optimum concentration for AgNPs production (Figure 1c).

### 3.3 UV‐Visible spectroscopy

**FIGURE 2:**
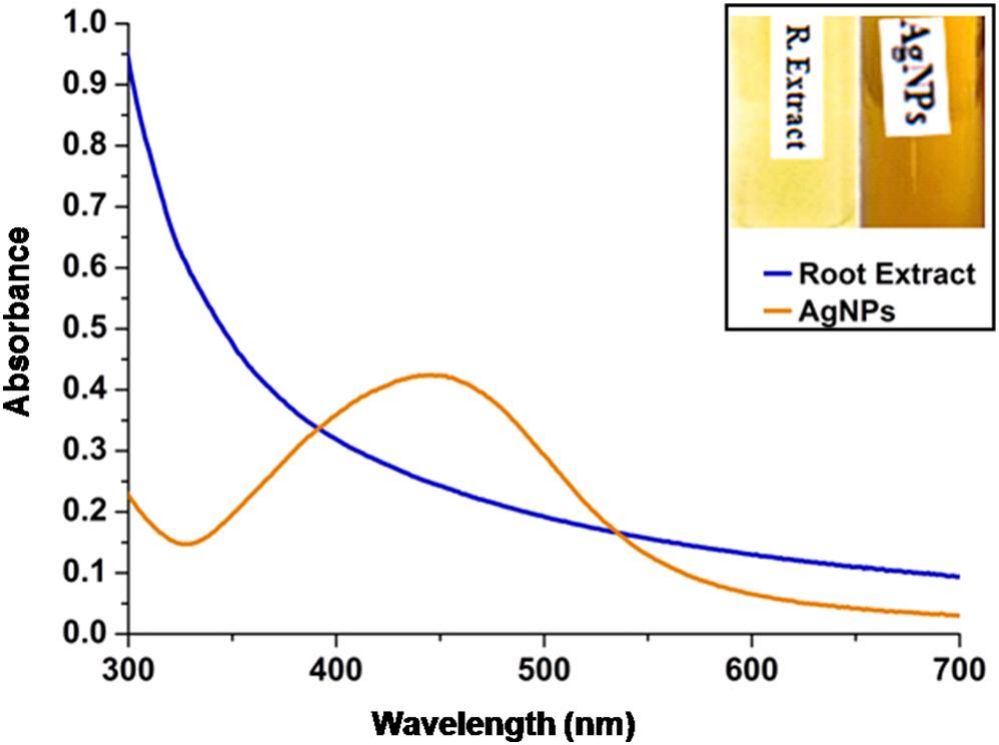
The UV‐Visible absorption spectra of AgNPs synthesized using root extract of M. dioica

The SPR peak at 445 nm confirmed the reduction of silver ions within 10 min (Figure 2). The dielectric constant, size, and shape of metal nanoparticles and their surrounding medium determine the width of surface plasmon absorption and frequency [38]. The intensity of the SPR band increased gradually as a function of the reaction time without change in the wavelength.

### 3.4 FT‐IR analysis of AgNPs

FT‐IR study was done to detect the functional groups which may belong to bioactive metabolites in the root extracts of *M. dioica* (Supplementary Figure 1a) acting as reducing agents for the synthesis of nanoparticles. The FT‐IR spectral data of nanoparticle revealed different absorption bands which are positioned at 3452, 2071, 1636, and 696 cm^−1^, in the region of 400 to 4000 cm^−1^ shown in (Supplementary Figure 1b) along with other low intensity bands. The absorption band at 3452 cm^−1^ (‐O‐H stretching) indicates the presence of alcohol and phenol groups in the root extract [12], 2070 cm^−1^ (‐C‐H stretching) indicates the alkane group, the strong bands at positions 1636 cm^−1^ (–C=O) and 696 cm^−1^ (‐C‐H) indicate the presence of the amide group and aromatic vibrations, respectively [31]. The bands corresponding to vibrational bonds such as ‐C=O, ‐O‐H, ‐C=H, and ‐C‐H may be due to the presence of derivatives of biomolecules such as enzymes, protein, triterpenoids, alkaloids, flavonoids, and sugars, which may act as reducing agents for the synthesis AgNPs.

### 3.5 XRD analysis of AgNPs

The XRD Analysis NPs of the *M. dioica* root extract revealed four different Bragg diffraction peaks positioned at 2*θ* values of 38.22, 44.40, 64.73, and 77.70° which is the characteristic future for nanoparticles. The crystalline nature of face centred cubic (fcc) structure of the AgNPs which is measure of purity is confirmed by the presence of peaks in the XRD pattern correlating to (*111*), (*200*), (*220*), and (*311*) planes. The diffraction peaks were more stable as per the standard database files of silver (JCPDS card No 04‐0783), indicating that the biosynthesized nanoparticle was crystalline in nature. The size of nanoparticles was determined as 11.2 nm from the XRD data by the Debye‐Scherrer equation (*d* = (*kλ*/*β* cos*θ*) [50] (Supplementary Figure 2).

### 3.6 TEM and SAED analysis of AgNPs

**FIGURE 3:**
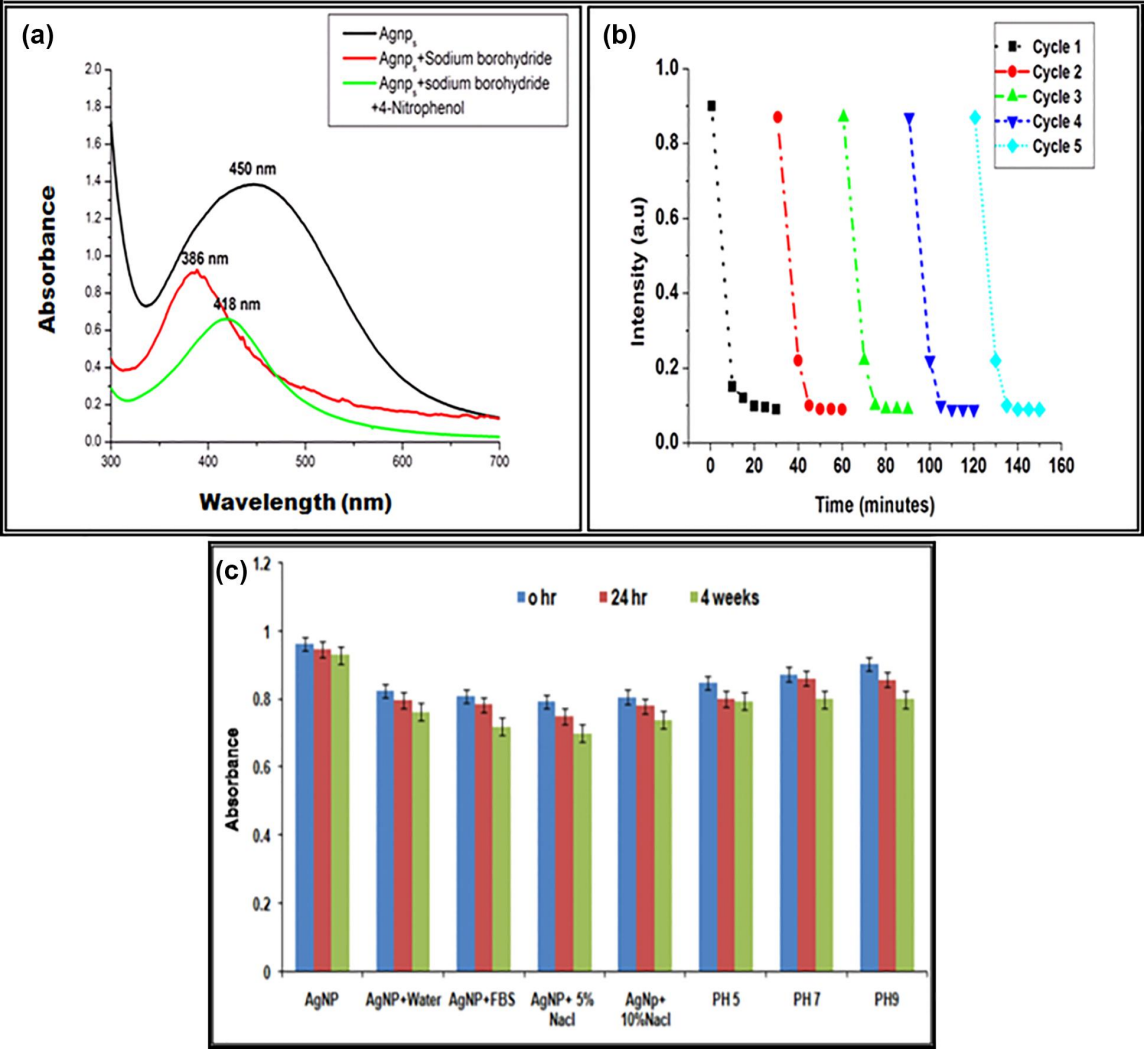
a) UV‐Vis spectrum of the reduction of 4‐NP by the silver nanoparticle. (b). Reuse and recycling of silver nanoparticle for the reduction of 4‐nitrophenol (c). In vitro stability studies of the AgNPs different biological fluids synthesized using the root extract Momordica dioica

The TEM images of nanoparticles synthesized using the root extract of *M. dioica* were given in Figure 3a. The green synthesized NPs were mostly spherical in shape with an average size of 13.2 nm and polydispersed. These results are similar to that reported by Kumar et al. for AgNPs synthesized using the Andean blackberry fruit extract [51]. The selected area electron diffraction (SAED) patterns of the AgNPs confirmed crystalline nature by showing four ring‐like diffraction patterns. This indicates that the biosynthesized NPs were pure. The diffraction patterns were assigned based on reference of the face centred cubic (fcc) structure of Ag. The four diffraction rings were observed in the SAED pattern are due to the reflections from (*111*), (*200*), (*220*) and (*311*) lattice planes of fcc Ag (Supplementary Figure 3b). The lattice planes of the AgNPs were evident by sharp Braggs reflection noted in the XRD spectrum.

### 3.7 Dynamic light scattering (DLS)

Zeta potential results revealed that the synthesized AgNPs are charged negatively with a potential of −22.3 mV. The general stability of nanoparticles was reported around a minimum charge of −30 mV [52]. The polydispersity index (PDI) showed a narrow size distribution of all the synthesized NPs evidenced by a value of PDI below 0.7 (Supplementary Figure 4a). The PDI index for AgNPs was 0.358 (Supplementary Figure 4b). These results clearly showed that the synthesized nanoparticles have a negative surface charge with a narrow size distribution.

### 3.8 Inductively coupled plasma atomic emission spectrometry (ICP‐AES)

The inductively coupled plasma atomic emission spectrometry is an analytical method used to confirm the type of metal based on the photon emission from excited atoms [53]. ICP‐AES was used to quantify the concentration of the Ag in AgNPs sample which was found to be 8.23 µg/ml.

### 3.9 In vitro stability catalytic studies of metal nanoparticles

The in vitro stability of the nanoparticles is a most important parameter for medical applications. The stability of green synthesized AgNPs was investigated in different solutions like foetal bovine serum, phosphate buffer, DPBS (pH 5.0, 7.0, and 9.0), and NaCl. After 24 h incubation of the synthesized AgNPs in the solutions, no major changes were detected in the UV‐Visible absorbance spectra of the AgNPs (Figure 3c). These studies suggested that the root extract (*M. dioica*) mediated AgNPs were highly stable in biological fluids and buffers. Because of their long‐term stability, these biogenic NPs can be ideal for use in drug delivery and other therapeutic applications [54]. The catalytic activity of the silver nanoparticles was estimated by the reduction of 4‐NP to 4‐aminophenol (4‐AP) by NaBH_4_ (Figure 3a) [55]. The AgNPs were separated from reaction system. The catalysis reaction again to study the reusability (Figure 3b), display five cycles of utilize of AgNP for reduction of 4‐nitrophenol. The catalytic activity of metal AgNPs did not reduce visibly even after the fifth cycle, which testify that the AgNP as a catalysis could keep strong catalysis property [56].

### 3.10 Antimicrobial activity of silver nanoparticles synthesized from the *M. dioica* root extract

#### 3.10.1 Minimum inhibitory concentration of AgNPs

The lowest concentration of AgNPs, at which the visible growth of the test microbes is totally absent, was considered as MIC. The antimicrobial activities of biosynthesized AgNPs against test pathogenic bacteria and fungal strain *C. albicans* were studied. The AgNPs showed relatively significant antimicrobial effect in the range of 3.125 to 6.25 µg/ml concentrations against the tested microorganisms compared to that of standard drugs such as ampicillin (3.125 µg/ml). The mechanism of action for antimicrobial potential of AgNPs was well studied [57]. The size of NPs is known to greatly influence their properties; the activity of nanoparticles increases with decrease in the size of the nanoparticle this is due to the increase in surface area per unit volume. Owing to their smaller size, the biosynthesized NPs showed large spectrum of specificity (based on TEM and DLS data). In addition, the biosynthesized AgNPs also showed good bactericidal and fungicidal activities. The biosynthesized AgNPs showed a good biofilm degradation potential by influencing ROS accumulation which induced the membrane damage in pathogens as explained later.

In the era of increasing MDR, development of multi‐drug resistance to different classes of antibiotics, is posing a challenge for treating many infectious diseases, thus leading to higher morbidity and mortality. NPs are considered as a reliable alternative to conventional antibiotics and seem to have a good potential to face the problem of bacterial MDR. The Gram‐positive bacteria contains a thick cell wall which is made of peptidoglycan layers containing linear polysaccharide molecules. These polysaccharides are linked by short peptides making them rigid structures which offer resistance to penetration of AgNPs compared to the Gram negative bacteria [58]. It was assumed that the high bactericidal activity of agents was attributed to the Ag^+^ ions released from AgNPs. Silver cations interact with the membrane and cause structural changes leading to increased permeability of cell wall or denaturation of membrane proteins of the bacteria resulting in cell death [59]. The binding of each Ag ions or nanoparticles to the cell membrane induces accumulation of protein precursors of the envelop. This results in the reduction of proton motive force. Jian‐Cheng et al. [60] explained the destabilization of outer cell membrane and rupture of plasma membrane by AgNPs which resulted in the reduction of intracellular ATP.

#### 3.10.2 Determination of the biofilm inhibition activity

**FIGURE 4:**
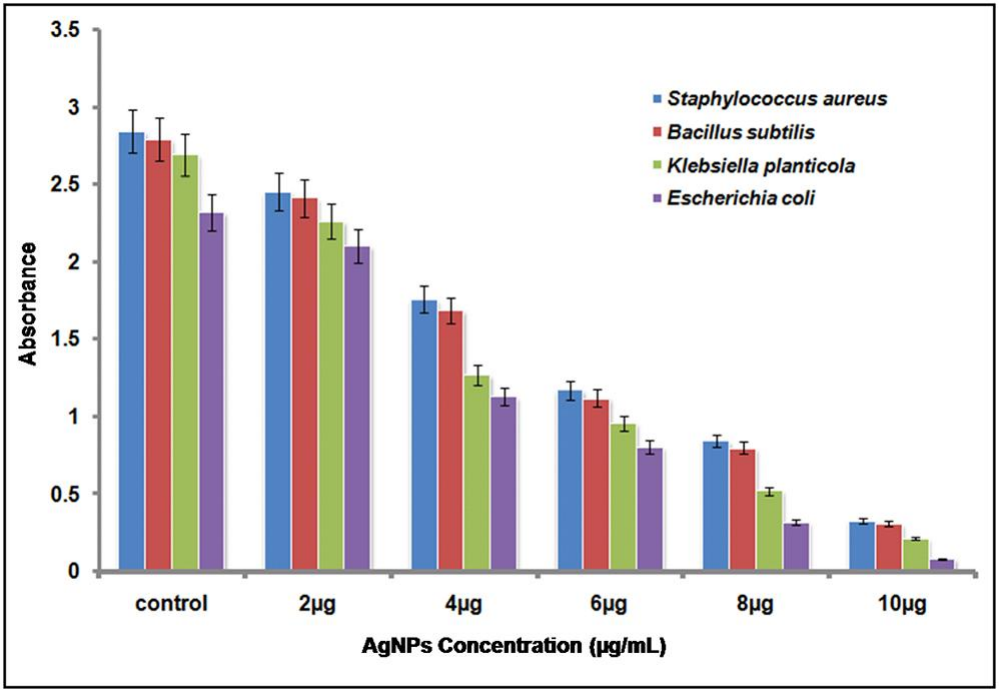
Biofilm inhibition by silver nanoparticles against S. aureus, B. subtilis, K. planticola and E. coli. All the experiment was performed in triplicates and the results are expressed as mean ± SD (*n* = 3)

In general, the anti‐bacterial resistance of bacteria is associated with the biofilm formation. The anti‐biofilm activity of green synthesized AgNPs assessed against different bacterial strains using crystal violet assay showed a concentration‐dependent reduction in biofilm biomass of pathogenic microbial strains. The increased resistance of the sessile cells to antibiotic treatment is mainly due to the biofilm formation. The consequences of biofilm inhibition studies recommend that the synthesized AgNPs were capable to inhibit the biofilm formation at the concentration of 10 μg/ml against *S. aureus*, *B. subtilis*, *K. planticola* and *E. coli* efficiently (Figure 4). Comparable effects were observed by the treatment of nanoparticles against *Staphylococcus pneumonia*, *P. aeruginosa*, *Staphylococcus flexneri*, and *S. aureus* [61]. Ansari et al. [62] reported in their study that colonies were grown in the absence of AgNPs and found that the organisms exhibited dry crystalline black colonies, suggesting the production of microbial exopolysaccharides, which is the prerequisite for the improvement of biofilm, but when the cultures were grown in the presence of AgNPs, no colonies were observed. Altogether, our data demonstrate that the AgNPs’ induced biofilm inhibition is more evident compared to AgNPs’ induced cell death suggesting different signalling mechanisms involved in cell survival and biofilm formation. However, many conventional synthetic methods are available for AgNPs synthesis, previous reports suggested that biologically synthesized nanoparticles were more active and showed more efficacies in terms of antimicrobial activity and minimal cytotoxicity potential. Thus, considering these advantages, *M. dioica* extract can be considered as a green resource for efficient synthesis of AgNPs.

#### 3.10.3 Killing kinetics of microbes

**FIGURE 5:**
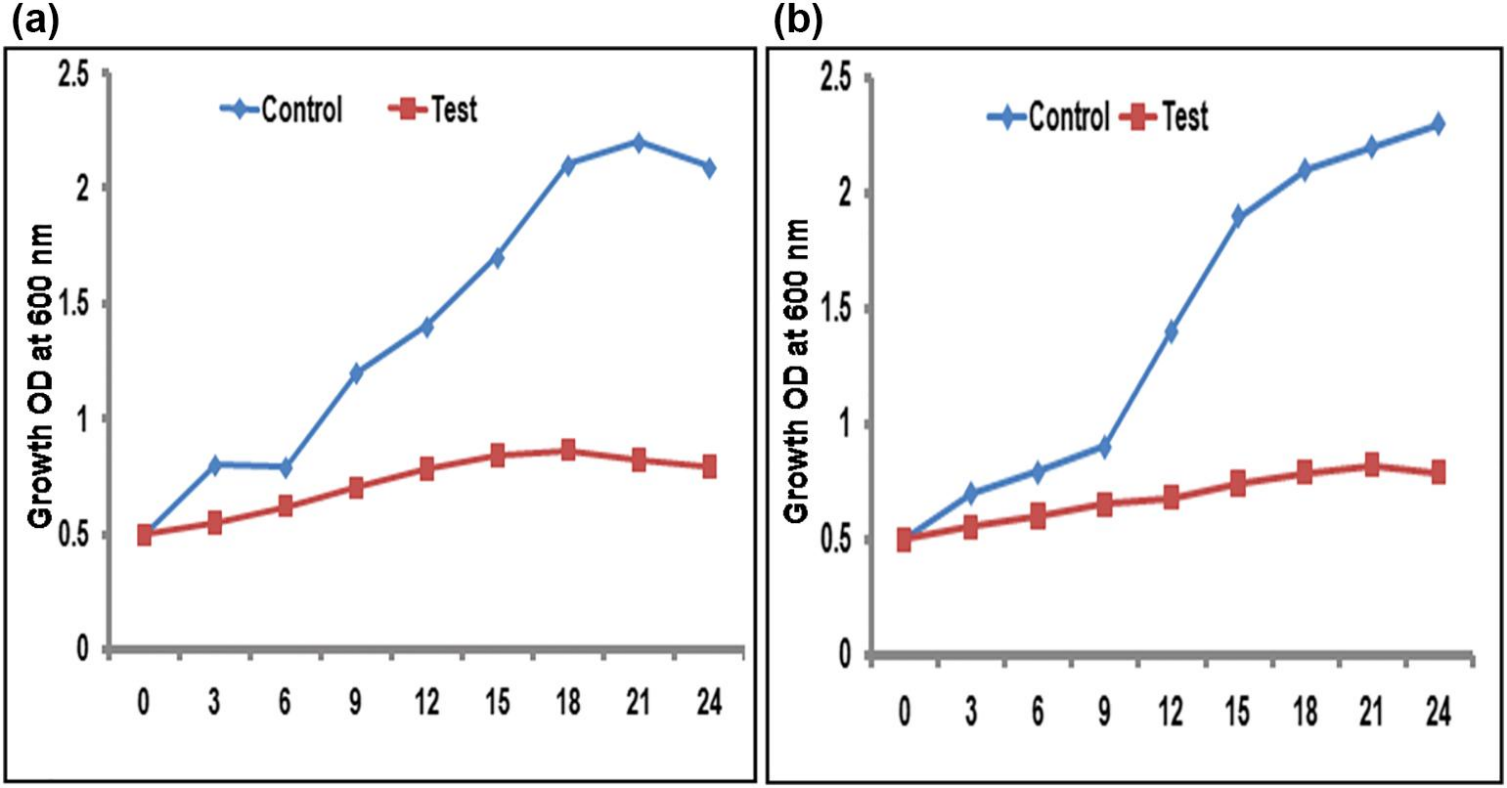
Growth kinetics of the (a) S. aureus (b) E. coli incubated with AgNPs synthesized using the M. dioica root extract. Each experiment was performed in triplicates and the results are expressed as mean ± SD (*n* = 3)

The growth kinetics of the microbes such as *E. coli* and *S. aureus* clearly showed the inhibition of microbial growth by the addition of AgNPs. The tested microbes such as *E. coli* and *S. aureus* were treated with suitable MIC concentration of both pathogens in MHB broth. After exposure to AgNPs, all the tested pathogens showed inhibition of growth within 3 to 4 h as compared to untreated cells. Our results showed that inhibition of all the tested bacterial strains, treated with AgNPs, were compared to the negative control (bacterial culture grown in absence of AgNP) [63]. This has been recognized to the reduction of microbial growth owing to the antimicrobial activity of AgNPs (Figure 5).

#### 3.10.4 Measurement of protein leakage.

**FIGURE 6:**
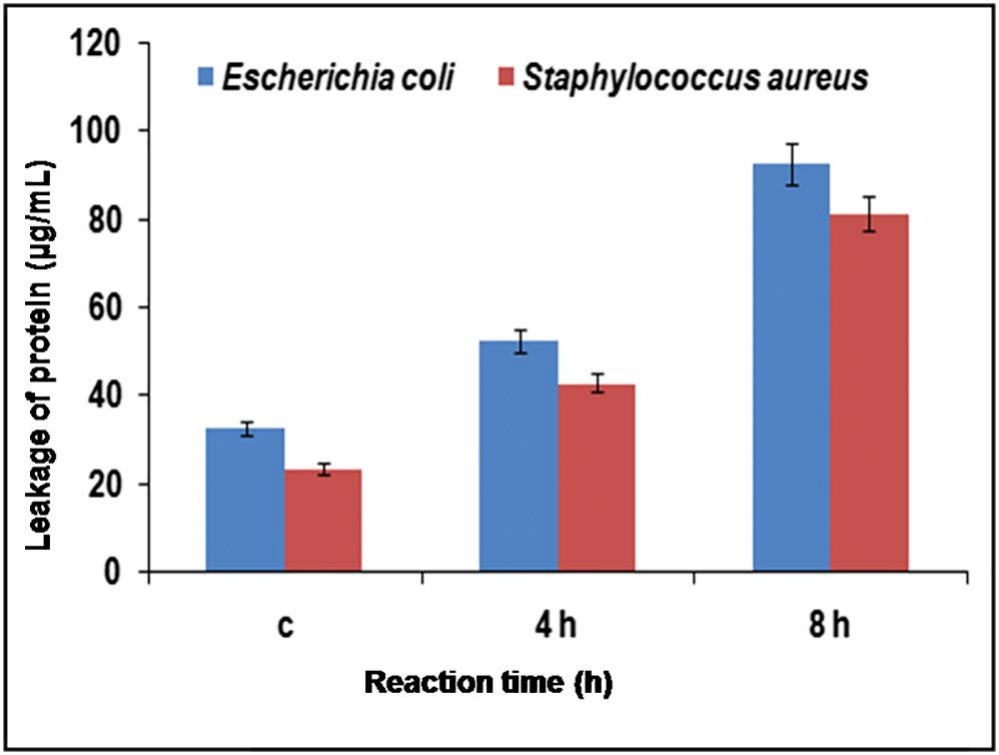
The effect of silver nanoparticles on protein leakage of bacterial cells. All experiments were performed in triplicates and the results were expressed as mean ± SD (*n* = 3)

The leakage of cytoplasmic proteins results in cell death. At the initial stage of experiment the observed protein leakage from both AgNPs treated and untreated cells were almost the same. After treating with AgNPs for 4 h, about 30–40 μg/ml protein leakage was observed. However, no protein leakage was observed in control cells. Similar results were reported for AgNPs treated *S. aureus* and *P. aeruginosa* [64]. Upon further incubation with AgNPs for up to 8 h, protein leakage from cells increased by twofold. It was observed that the AgNPs treated *E. coli* cells lost higher amount of protein (92 μg/ml) compared to *S. aureus* (81 μg/ml) after 8 h of treatment with AgNPs (Figure 6) which may be due to difference in cell wall thickness.

#### 3.10.5 Nucleic acid leakage assay

**FIGURE 7:**
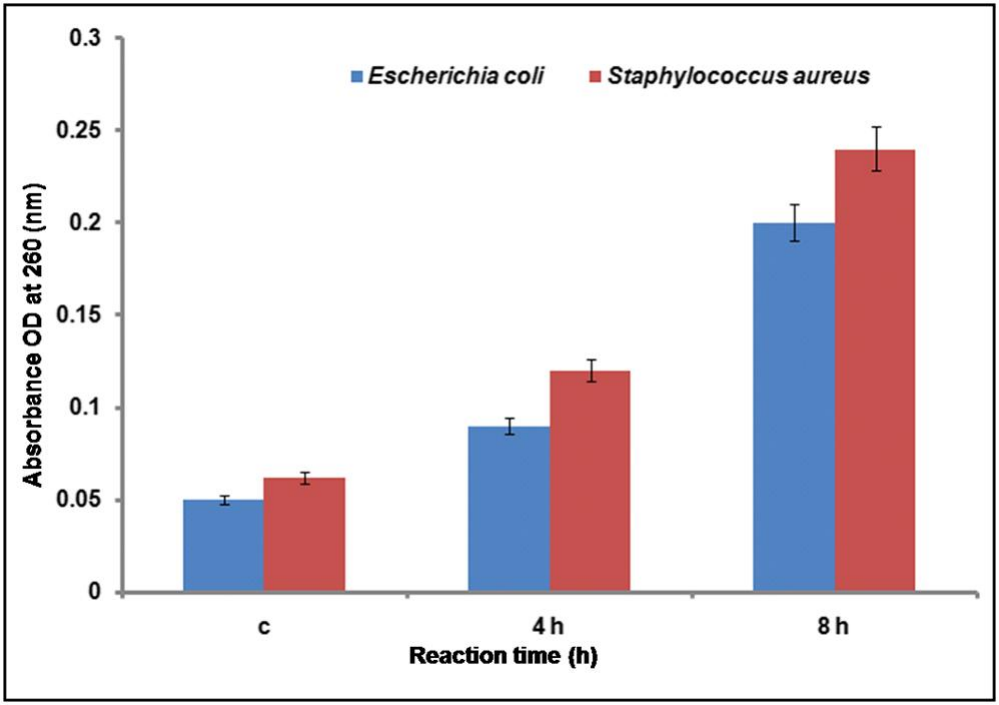
The effect of AgNPs on bacterial cells showed the highest amount of nucleic acid leakage than the control cells. Each experiment was performed in triplicates and the results were expressed as mean ± SD (*n* = 3)

The cell‐free filtrates were analysed for the presence of nucleic acids leaked from cells due to AgNPs exposure by measuring OD at 260 nm. Nucleic acid leakage was not observed initially in cells treated with nanoparticles as well as the control cells (Not treated with AgNPs). Although AgNPs’ treated cells showed significant increase in nucleic acids after 4 and 8 h of incubation in the cell‐free filtrate, this condition was caused due to the leakage by the AgNPs. *S. aureus* was relatively more affected by AgNPs treatment compared to *E. coli* which is evident by the high level of nucleic acid release observed (Figure 7). The cell free filtrates of untreated microbial cells contained lower levels of nucleic acids than that of AgNPs treated cells [65].

#### 3.10.6 ATP leakage assay

**FIGURE 8:**
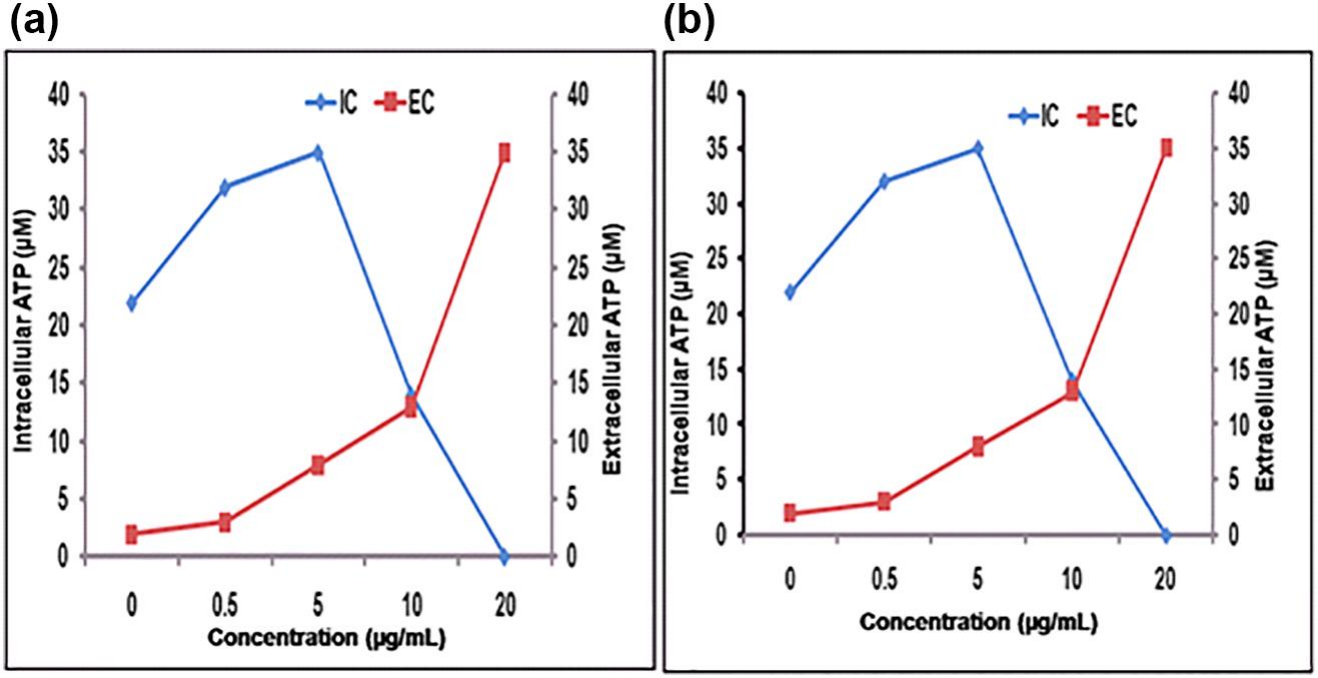
The intracellular (IC) and extracellular (EC) ATP levels of (a) S. aureus and (b) E. coli treated with different concentrations of AgNPs. All the experiments were performed in triplicates and the results were expressed as mean ± SD (*n* = 3). The results were statistically significant (*p* < 0.001)

The release of intracellular components especially ATP is an important parameter for assessing cell death. ATP bioluminescence assay is used to know if cell membrane perturbation was associated with cell death. The AgNPs treated pathogenic strains (*S. aureus* and *E. coli*) showed release of intracellular ATP. In our study, the microbes were treated with AgNPs of specific concentration, which was not their respective MIC value. The concentration of intracellular ATP decreased fast and almost reached zero for the tested pathogens after a few minutes at 20 μg/ml concentration. This corresponded to the increase in the extracellular ATP concentration (Figure 8). For the survival of microbial cells and normal physiological functions a minimum concentration of 5 μM intracellular ATP is required. The results showed that the intracellular ATP levels decreased below the min survival concentration suggesting that the AgNPs are potential in inducing ATP leakage which is an important indicator for cell death [66].

#### 3.10.7 Measurement of intracellular potassium levels

**FIGURE 9:**
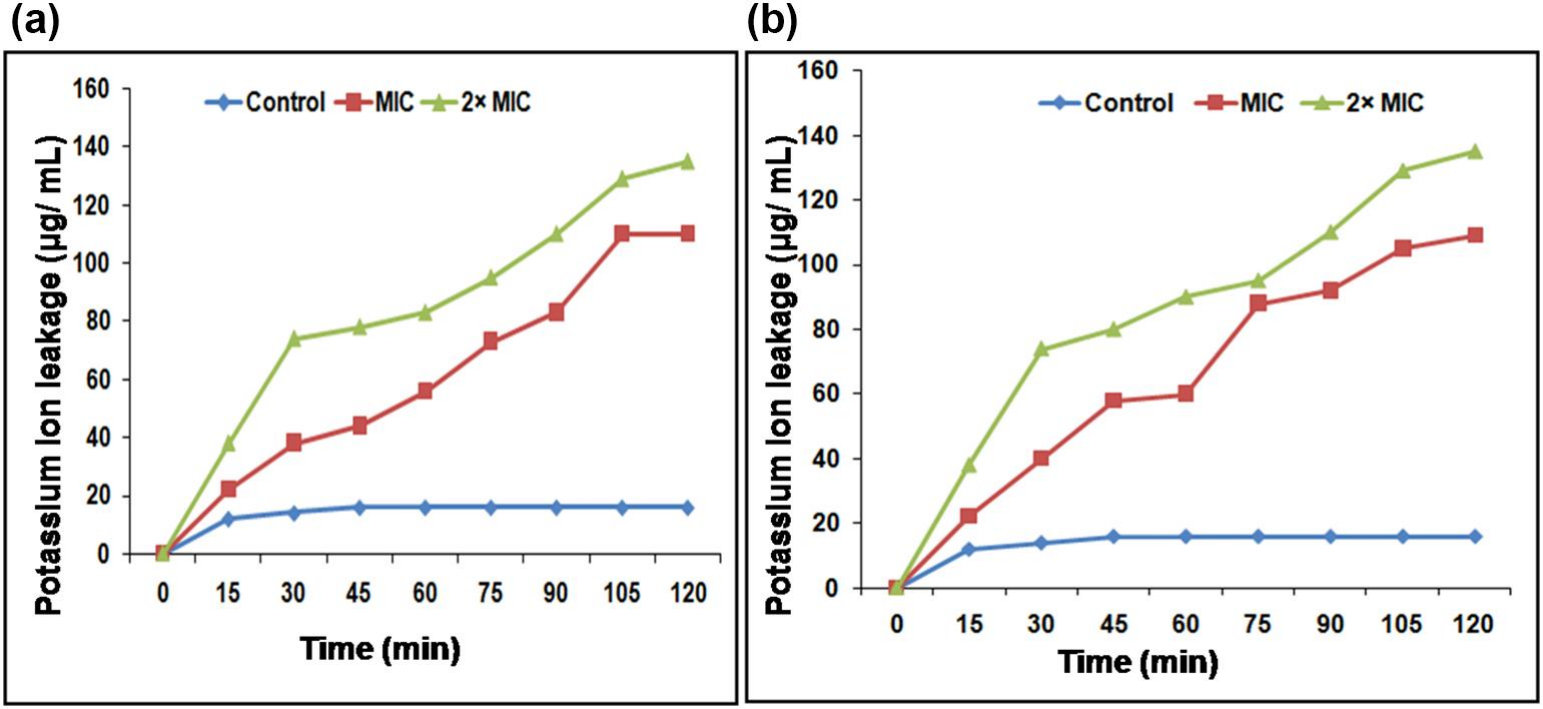
Measurement of intracellular potassium in (a) S. aureus and (b) E. coli treated with different concentrations of AgNPs. Each experiment was performed in triplicates and the results are represented as mean ± SD (*n* = 3)

In the current study, intracellular K^+^ leakage was observed in bacterial cells treated with AgNPs, when compared to controls. Intracellular K^+^ leakage was observed after 15 min of AgNPs treatment which increased up to 100 min for AgNPs treated cells of *S. aureus* and *E. coli* at respective MIC levels. Increasing the MIC level of AgNPs by onefold resulted in an increase in the leakage of K^+^ in both organisms. The AgNPs exposed bacteria showed maximum loss of intracellular K^+^ within 2 h where more than 60% loss occurred within 1 h of incubation (Figure 9). This may be due to the induction of membrane damage by AgNPs [67].

#### 3.10.8 Measurement of intracellular ROS

**FIGURE 10:**
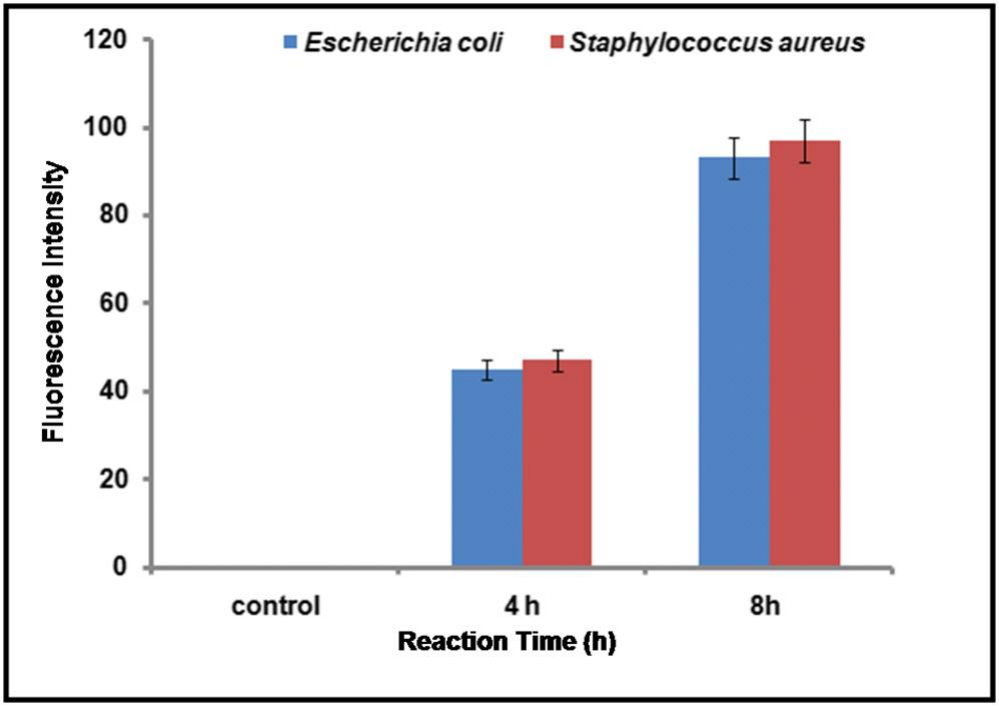
Formation of ROS in E. coli and S. aureus cells exposed to AgNPs. Graph showing ROS generation using DCFDA in control, hydrogen peroxide (H_2_O_2_ – 1 mM). All the experiments were repeated four times and the data are represented as mean ± SD (*n* = 4). The results are statistically significant (*p* < 0.001)

In the present study, ROS concentration was measured by the DCFDA method. Initially ROS was detected after 4 h of incubation in AgNPs‐treated S*. aureus* and *E. coli*. After 8 h of incubation, the concentration of ROS in both organisms increased 0.9 fold (Figure 10). The ROS was not observed in the control sample (not treated with AgNPs). Rastogi et al. [45] studied the gum Kondagogu synthesized AgNPs where a 2.0 fold increase in ROS was observed in AgNP‐treated *S. aureus* cells. Previous researchers have reported that ROS naturally occur in both intracellular and/or extracellular spaces [68]. These results suggest that AgNPs can influence the generation of ROS in water, which can cause damage to bacterial cell walls, proteins, and intracellular components. The antimicrobial activity of nanoparticles is associated with the free radical generation. Increased levels of ROS under some conditions increase the oxidative stress in bacterial cells. Oxidative stress leads to damage of cell membrane, in addition to protein and DNA degradation, and damage to components comprising cellular respiratory systems.

## 4 CONCLUSIONS

In this study, we synthesized AgNPs using an eco‐friendly, cost effective, and non‐toxic approach using the *M. dioica* root extract. The green synthesized AgNPs particles were uniform in shape with an average size of 13.2 nm. In this study, the biosynthesized AgNPs showed potent antibacterial activities against *S. aureus, B. subtilis, E. coli, P. aeruginosa, K. planticola* and *C. albicans* when compared with standard drugs like ampicillin and miconazole. Other mechanistic studies revealed that the nanoparticle induces oxidative stress which inhibits the growth of pathogens. The observed experimental results showed leakage of the intracellular content suggesting that AgNPs could cause membrane damage‐mediated microbial cell death. The detailed mechanistic studies provided a plausible explanation for the elusive cell death mechanism of AgNPs in *S. aureus* and *E. coli*. Hence, the phyto‐fabricated synthesized NPs can be used as prospective antimicrobial mediators in the near future.

## CONFLICT OF INTEREST

The authors declare that there is no conflict of interest.

## Supporting information

Supporting Information S1
