# Supplementary material for: Phyto‐fabricated silver nanoparticles inducing microbial cell death via reactive oxygen species‐mediated membrane damage
Source: IET Nanobiotechnol. 2021 Apr 21;15(5):492–504. doi: 10.1049/nbt2.12036 (PMC8675829; doi:10.1049/nbt2.12036)
Supplement: Supplementary file 1 — Supporting Information S1 [file NBT2-15-492-s001.docx]

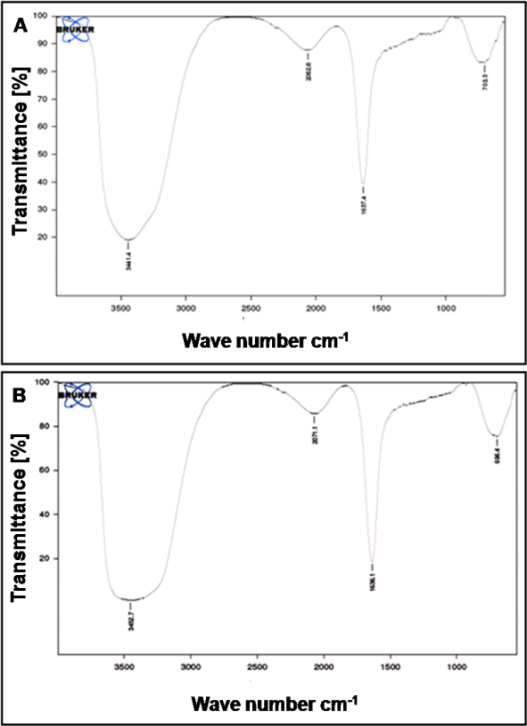


**Supplementary Fig. 1:** FT-IR spectra of the a) *M. dioica* root extract and b) AgNPs synthesized using *M. dioica* root extract

**3.5 XRD Analysis of AgNPs**

The XRD Analysis NPs of the *M. dioica* root extract revealed four different Bragg diffraction peaks positioned at 2θ values of 38.22, 44.40, 64.73 and 77.70 degrees which is the characteristic future for nanoparticles. The Crystalline nature of face centered cubic*(*fcc*)*structure of the AgNPs which is measure of purity is confirmed by the presence of peaks in the XRD pattern correlating to (*111*), (*200*), (*220*) and (*311*) planes.  The diffraction peaks were more stableas per the standard database files of silver (JCPDS card No 04-0783), indicating that the biosynthesized nanoparticle were crystalline in nature. The size of nanoparticles was determined as 11.2 nm from the XRD data by the Debye-Scherrer equation (d = (kλ / β cosθ) [52], (Supplementary Fig. 2).


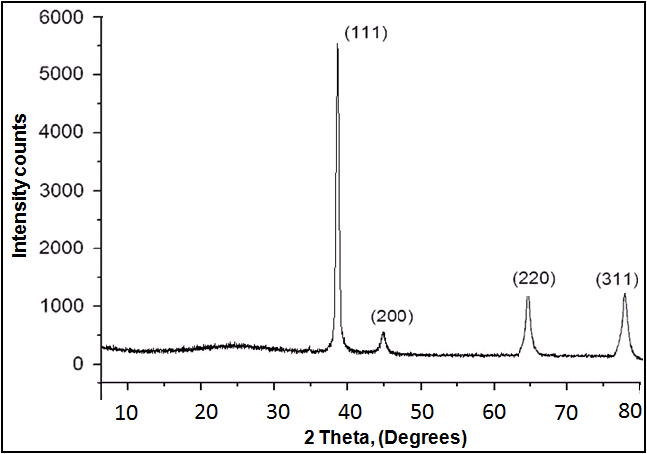


**Supplementary Fig. 2:** X-ray diffraction (XRD) spectrum of biosynthesized AgNPs using *M. dioica* root extract.

**3.6 TEM and SAED Analysis of AgNPs**

The TEM images of nanoparticles synthesized using root extract of *M. dioica* were given in figure. 3A. The green synthesized NPs were mostly spherical in shape with an average size of 13.2 nm and polydispersed. These results are similar to results reported by Kumar B et al, (2017) for silver nanoparticles synthesized using Andean blackberry fruit extract [53]. The selected area electron diffraction (SAED) patterns of the AgNPs confirmed crystalline nature by showing four ring-like diffraction patterns. This indicates that the biosynthesized NPs were pure. The diffraction patterns were assigned based on reference of the face centered cubic (fcc) structure of Ag. The Four diffraction rings were observed in the SAED pattern are due to the reflections from (*111*), (*200*), (*220*) and (*311*) lattice planes of fcc Ag (Supplementary Fig. 3B). The lattice planes of AgNPs were evident by sharp Braggs reflection noted in the XRD spectrum.


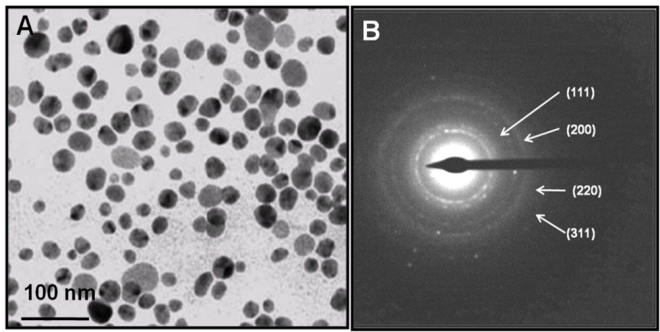


**Supplementary Fig. 3:** (A) TEM micrographs of AgNPs with an average size of 13.2 nm polydispersed on an amorphous carbon-coated copper grid. (B) Selected area electron diffraction (SAED) pattern of AgNPs. The Miller indices were assigned to the diffraction rings in accordance with the face-centered cubic (fcc) lattice of Ag.

**3.7 Dynamic light scattering (DLS)**

Zeta potential results revealed that the synthesized AgNPs are charged negatively with a potential of -22.3 mV. The general stability of nanoparticles was reported around a minimum charge of -30 mV [54]. The polydispersity index (PDI) showed a narrow size distribution of all the synthesized NPs evidenced by a value of PDI below 0.7 (Supplementary Fig. 4A). The PDI index for AgNPs was 0.358 (Supplementary Fig. 4B). These results clearly showed that the synthesized nanoparticles have negative surface charge with a narrow size distribution.


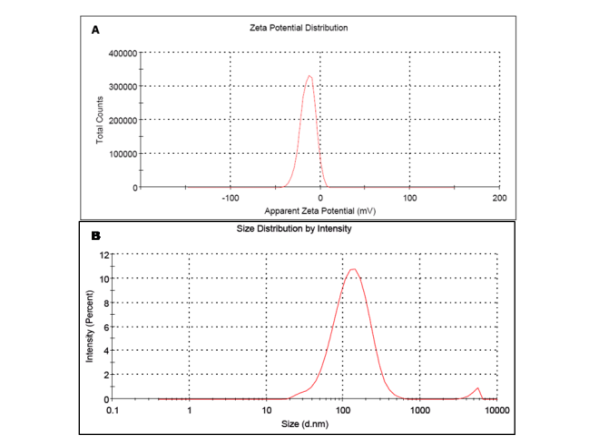


**Supplementary Fig. 4:** (A) Zeta potential analysis of AgNPs and (B) Particles size distribution of biosynthesized AgNPs.

**3.8 Inductively coupled plasma atomic emission spectrometry (ICP-AES)**

The Inductively coupled plasma atomic emission spectrometry is an analytical method used to confirm the type of metal based on the photon emission from excited atoms [55]. ICP-AES was used to quantify the concentration of the Ag in AgNPs sample which was found to be 8.23 µg mL^-1^.

**3.9 In vitro stability catalytic studies of metal nanoparticles**

The *in vitro* stability of the nanoparticles is a most important parameter for medical applications. The stability of green synthesized AgNPs was investigated in different solutions like fetal bovine serum, phosphate buffer, DPBS (pH 5.0, 7.0 and 9.0), and NaCl. Subsequent to 24 h incubation of synthesized silver nanoparticles in the solutions, no major changes were detected in the UV-visible absorbance spectra of the AgNPs (Fig. 3c). These studies suggested that the root extract (*Momordica dioica*) mediated silver nanoparticles were highly stable in biological fluids and buffers. Because of their long term stability, these biogenic NPs can be ideal for use in drug delivery and other therapeutic applications [56]. The catalytic activity of silver nanoparticles was estimated by the reduction of 4-NP to 4-aminophenol (4-AP) by NaBH_4_ (fig. 3a) [57]. The AgNPs were separated from reaction system. The catalysis reaction again to study the reusability, (fig 3b) display five cycles of utilize of silver nanoparticle for reduction of 4-nitrophenol. The catalytic activity of metal AgNPs did not reduce visibly even after the fifth cycle, which testify that the silver nanoparticle as a catalysis could keep strong catalysis property [58].
